# Physical Education of Women

**Published:** 1856-07

**Authors:** 


### THE PHYSICAL EDUCATION OF WOMEN.*

THE first work upon our list treats of those principles of physical and mental education which have since been widely popularised by Andrew and George Combe. Whatever may be thought of the special claims of phrenology to be regarded as a science, there is no doubt that the founders and promulgators of that system, which regards all mental and many physical operations as the result of certain conditions of the brain, introduced virtually a new element into medicine, and into many of the most knotty problems of social life. The divine and the poet may, indeed, consider that the works of Gall and Spurzheim, or the clear impressive *Constitution of Man*, omit to state fairly the mysteries of human life, and slur over rather than present any solution of "the riddle of the painful earth". But these works nevertheless introduce an immense body of facts and of insurmountable arguments, which have gradually worked into the thoughts of thinking men of all denominations and habits of mind, and which must henceforth form the basis of all philosophical systems, and of all practical legislation. Calvinists and Methodists are forced to recognise the importance of sanitary reform; Draco himself, had he lived in our day, must necessarily have paid attention to the limits of sanity, and to the intimate connexion between the asylum, the prison, and the gallows; and not the least among the triumphs of inductive science is that change in public opinion which has taken women out of the semi-angelic, semi-slavish, position which they once occupied in the ideas of the other sex, and reduced or exalted them to the level of a constituent part of the human race, with bodies and brains amenable to the same general laws as those of

---

* SPURZHEIM, J. C., M.D. A View of the Elementary Principles of Education, founded on the Study of the Nature of Man.

BLACKWELL, Elizabeth, M.D. The Laws of Life, with Special Reference to the Physical Education of Girls. New York.

Report of the Commissioner appointed to Inquire into the Condition of the Frame-Work Knitters. London: 1845.

Report of the Commissioner appointed to Inquire concerning Bleaching Works, etc. London: 1855.

men, and lying under the same stern necessity which declares "as thou sowest, so shalt thou reap". The fact having been gradually made clear to the majority of minds, that the fair and comparatively delicate frames of the "softer sex" are of the earth earthy, even as the flowers themselves, and bear the ordinary human relations to chemical and mechanical laws, it follows that exercise, cold bathing, loose healthy clothing, sound sleep, and a due measure of mental occupation, begin to be recognised as essential elements in the physical education of girls, whether they are considered as beings created for manifold use and enjoyment of their own natures, or simply in regard to the one physical duty which they owe to the next generation—that of bearing healthy children.

The excellent American work which comes second on our list, is the legitimate offspring of the school of thought to which the first belongs. Its author is of English birth, but was educated in America, and there conceived the desire to study medicine, regarding it as an eminently suitable branch of science for the attention and practical application of women. She took a regular medical degree at Geneva College, New York State, and coming to Europe, she spent some time in the hospital of La Maternité in Paris, after which she was permitted to study at St. Bartholomew's for some months, and then, returning to America, she commenced practice in New York. Her progress has been slow but sure, and she is at the present time in possession of increasing confidence and respect, not only from female patients, but from men of ability and standing in New York, who have accepted both herself and her principle as a marked fact and a growing truth of the time.

*The Laws of Life* formed the matter of a series of lectures delivered to a class of ladies during the spring of 1852; they are presented as "outlines of truth, and as indications of the right method of education, rather than as a full discussion of the subject." The lectures are six in number, and are thus entitled: Introductory; General Laws; The Organic Life; The Related Life; Criticism; Reform.

In a small volume under two hundred pages, a philosophical rather than a detailed scientific view of the subject is alone possible; and the English reader may think that the importance of health is by this time a tolerably well established fact, and does not need enforcement by such a mass of argument and illustration. The key-note of the book is the simple deduction, that "the perfect state of the organic life, of digestion, nutrition, innervation, and all the secretions, is the foundation stone of our ideal; and that we might as well attempt to build a marble edifice on rotten arches, as strive for perfection with a disordered stomach or weak nerves." But it needs very slight acquaintance with American literature, to detect the fact that the standard of robust health among the female part of the nation is very low, both in practice and in theory. Delicacy of frame is confounded with delicacy of nature—the one being supposed to be the outward expression of the other; and Elizabeth Blackwell says, that "there is no nation in the world where so earnest an attempt is made to cultivate the mind, with so complete a neglect of all the physical necessities of the child, as in the United States—and nowhere is the health of the women so feeble—and this weakness is on the increase." Her English reader, to whom such an assertion regarding the condition of the majority of the Anglo-Saxon race cannot be uninteresting or unimportant, may well ask himself whether English women, more robust and of healthier habits as they undoubtedly are, are as yet anything like up to the mark of a satisfactory physical development; and whether, in the great increase of intellectual cultivation, and the pursuit of various arts and professions by the sex, there is not much danger of serious evil arising from sedentary education and life? The school and the college give to the future lawyer or clergyman something like a firm basis of bodily strength; the cricket-ground, the boat-race, the pedestrian excursion, find no parallels in the early training of girls. If teachers possess the sense and the kindness to ensure active play in the open air to their little pupils, the stimulus ceases as soon as the days of tuition are over; and young things of sixteen or seventeen find themselves suddenly planted in the life of women, with no livelier exercise than a walk, or, at best, the dance in a heated ball-room, when they ought to be asleep. It is true that the practical difficulty of securing healthy exercise of various kinds to young women is not an easy thing in the present state of the world and of public opinion, but it is not impossible; and it must be done, or the physical evils incident to the class of women who are removed from the beneficial necessity of making beds and sweeping stairs, and whose high mental cultivation is every year increased and urged on by all that masters, emulation, and the ambition of parents can suggest, or by the requirements of professions in which they are forced to contend with men for the winning of daily bread, will in the end lead to utter degeneracy. It surely would not be impossible, for instance, to arrange some kinds of gymnasia in all large towns, where young girls might find such exercise as would be suitable to their powers, and which might preserve to them for a few more years somewhat of the suppleness and activity of childhood.

Regarding the true physical powers of the female sex, the greatest errors prevail. The degree of healthy exercise possible and suitable to women and girls is, indeed, so far above what is usually imagined, that it is worth while to dwell upon a few facts of female labour as undertaken by the lower classes of women in England. The *Report* of the inquiry into the condition of the operatives engaged in bleaching works, speaks of women and boys working fourteen, fifteen, and sixteen hours a-day, and of "the pains in the limbs directly referrible to the many hours that they are on their feet, and to the almost constant and sometimes rapid movement required." The *Report* also enters into various details, suggests that the hours should be limited to ten, and, "considering the fatiguing nature of much of the work of other descriptions in which both females and boys are engaged in the English bleach-works, especially" recommends a longer period of rest at meals. Pitiful are the statements of some of the young girls; and one woman, who had worked for twenty years, speaks of the "little ones falling asleep standing to their work." She says, "We start at half-past six in the morning, and leave off at eleven at night. This has been going on for two years. On Friday night a set comes on at ten o'clock, and works till Saturday at five. The set that comes on Saturday morning at six works till Saturday night at twelve; they start again at one o'clock on Sunday night. This has been so for two years, all the time that I have been here. We have in this room nine women and twenty-one girls, nine of whom are under thirteen. All these work the same number of hours. All the girls are always complaining of their legs aching and their feet being sore, and some are frequently off from illness; there are always one or two off in consequence of long hours and working at night. I have talked to them many a time about the hours being shortened like the factories. They wish that time would come." We learn of the framework knitters that John Geary, of Ansted, thinks that "The thing is quite inconsistent with reason that a woman should ever work in a frame at all; it is too heavy and too laborious for any female to work in, though there is a difference in the strength of women as well as in that of men;" also that the hours are as long as those of men, and generally from fifteen to eighteen, "very few less than fifteen, I should think." "'At what age do the young women commence working in the frames?'—'As soon as they can; the same age as boys, in a manner.'" And we learn on another page, that the peculiar characteristic of the stocking-frame, "of having escaped the propelling and invincible power of steam, has been ascribed to the fact that the varied movements of the body, hands, and legs, which are each called into action in the working of a stocking-frame, are all necessarily mainly regulated and guided by the eye."

Thus we find our women of the lower classes undergoing prolonged physical labour to an exhausting and utterly injurious degree, while the fine ladies of our upper ranks cannot walk five miles to save their useless and effeminate lives.

In a little work entitled *Health, Disease, and Longevity*, by Lionel John Beale, are some curious facts concerning extreme age in either sex; and we find record of a Mrs. Jones, of Cambridge workhouse, who died at the age of 125, enjoying her health and senses to the last; also of Mary Rogers, aged 118, who lived the last sixty years on vegetables. Mary Jenkins, 110, Clothworkers' almshouses, London, never had any illness, and died suddenly. Margaret Patten, 137, St. Margaret's workhouse, always enjoyed good health till a few days before death; in many years she chiefly subsisted on milk. Mary Wilkinson, 109, Ronaldkirk, York, when young, walked seven times to London and back; at the age of ninety, she buckled a keg of gin and a quantity of provisions on her back, and reached London in five days and three hours.

Here are examples, on the one side, of immense and harmful physical labour; on the other, of vigorous and long life; one proving directly, the others indirectly, how much exertion the female frame is capable of enduring, and how large might be the range of medium powers, were due attention paid to the physical education of the sex. To this end it is necessary that all false ideals as to the constituent elements of real grace and beauty of womanhood be exploded. They lie at the root of much of that enervated habit of life which has lowered the whole physical tone of the women of the United States; nor is Europe free from a tinge of the Chinese feeling, which desires to see a beautiful woman "as a swaying willow". The world is not willing to trust to the inherent softness and delicacy (we use the word in an artistic sense) of a woman's person, and has an idea that the natural lilies and roses of a lovely complexion are rendered daintier by sedentary habits and careful seclusion from the air. It will not recognise the artistic truth that the supreme beauty of anything, tree, flower, animal or human creature, can only consist with its perfect development; that the finish of Nature is infinitely more elaborate than the extremest finish of art. Once admitted, this law would sweep to the winds all those efforts to improve nature and gild refined gold, which are nowhere so absurd as when they interpose to mar the noblest work of Nature, by stays, tight shoes, false hair for the matron's brow, and cosmetics to imitate the inimitable hues of girlhood. Nature herself secures her own unities of time and place; confounds not the characteristic graces of different ages and different conditions. Art plays tricks with each essential charm of youth and age, and sacrifices in the struggle even more than beauty—the natural vigour of good health. Could mankind once be induced to trust wholly to the perfection of nature, a totally new system of physical education for the female sex would prevail. "Till then", to conclude in the expressive language of Spurzheim, "education will produce comparatively little effect. Man is the disciple of Nature, and he must submit to the determined sway which prevails in her government. He must submit himself to her; for he errs the moment he ceases to observe and begins to excogitate. The construction of a system of education cannot be a creative but an imitative process, which must be founded only on the lessons of experience. Here, as in the cultivation of every other science, it is not by the exercise of a sublime and speculative ingenuity that man arrives at truth, but it is by letting himself down to simple observation,—by rejecting equally the authority of antiquity and of eminent contemporaries, when in opposition to nature; by sacrificing every consideration that opposes the evidence of observation, and its legitimate and well-established conclusions; by being able to renounce all the favourite opinions of infancy, the moment that truth demands the sacrifice; in short, by following only the lights of observation and induction."

